# What Computational Simulations Tell Us about Cryptochromes in Magnetoreception

**DOI:** 10.1021/acs.biochem.6c00516

**Published:** 2026-10-21

**Authors:** Mariastella Cascone, Lorenzo Cupellini, Benedetta Mennucci

**Affiliations:** † Scuola Normale Superiore, Piazza dei Cavalieri 7, 56126 Pisa, Italy; ‡ Dipartimento di Chimica e Chimica Industriale, 9310 Università di Pisa, Via G. Moruzzi 13, 56124 Pisa, Italy

## Abstract

The ability of migratory birds to orient using the Earth’s magnetic field has been proposed to rely on a radical-pair mechanism operating within Cryptochrome 4, a blue-light flavoprotein. In this perspective, we review how computational approaches have shaped our molecular understanding of cryptochrome-based magnetoreception. We organize the discussion around the three steps that connect light absorption to a cellular signal: the photochemical step, in which light absorption by FAD drives a hole-hopping electron-transfer cascade along a conserved tryptophan tetrad; the spin-dependent step, in which the resulting radical pair interconverts between singlet and triplet states under the influence of the magnetic field; and the structural step, in which this transient information is converted into a long-lived signaling state. The early electron-transfer events are now described with growing confidence and appear conserved across avian orthologs, although the relative stability of the final radical-pair states remains debated. The spin-dependent step remains more problematic: once the fluctuations of the hyperfine couplings and a realistic number of nuclear spins are included, the predicted magnetic sensitivity at geomagnetic field intensities becomes very small, and explaining the magnitude of the effect is still a central open problem. The postactivation conformational dynamics, involving intrinsically disordered regions, remains the principal challenge. We suggest that atomistic multiscale modeling, integrated with artificial intelligence-based approaches, has become indispensable for connecting the conserved photochemistry to this downstream process.

## Graphical abstract

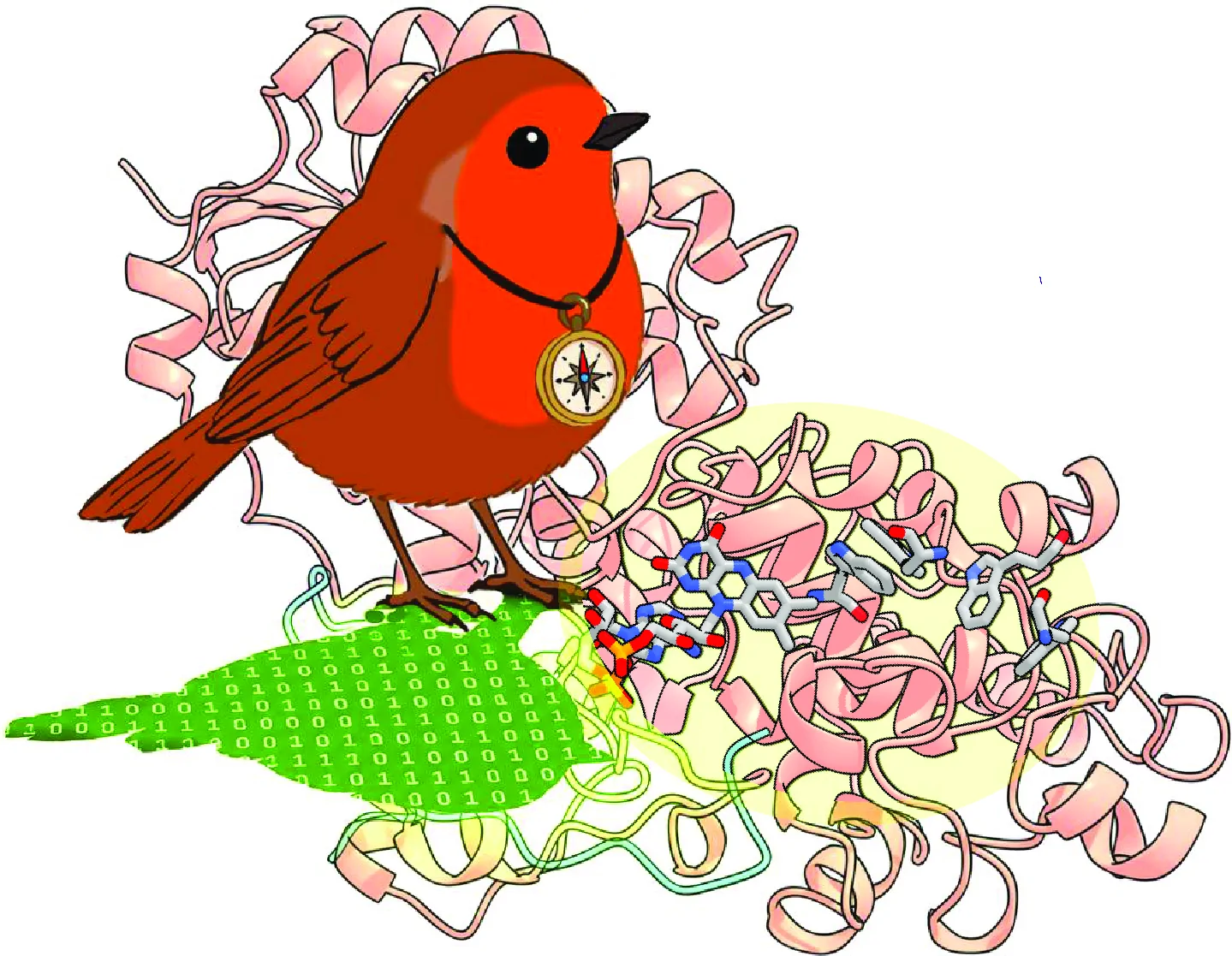

## Introduction

For more than 50 years, the magnetic compass sense of migratory birds has attracted considerable scientific interest, owing to its fundamental role in orientation and navigation during migration.^1,2^ Following the pioneering theoretical studies of Schulten and co-workers,^3,4^ cryptochromes have emerged as the leading molecular candidates for avian magnetoreception.

Cryptochromes (Crys) are blue-light photoreceptors that are widely distributed across different kingdoms of life.^5^ Together with photolyases, they constitute the Cryptochrome/Photolyase Family (CPF), a large protein superfamily that also includes CRY-DASH proteins, whose members are found in organisms ranging from *Drosophila* and *Arabidopsis* to cyanobacteria and humans.^6,7^ Phylogenetic analyses indicate that cryptochromes have evolved along distinct evolutionary pathways in different organisms^8^ and, although CPF proteins share significant structural similarities, their biological functions have diversified considerably. Photolyases and CRY-DASH proteins primarily act as DNA-repair enzymes, catalyzing the light-driven repair of UV-induced DNA damage.^9^ In contrast, cryptochromes have evolved to perform a broad range of biological functions, including circadian rhythm regulation, transcriptional control, photomorphogenesis, flowering-time regulation in plants, and magnetoreception in animals.^10–13^

Despite their functional diversity, cryptochromes display a highly conserved overall architecture (see Figure 1). The core of the protein is the Photolyase Homology Region (PHR), which is structurally similar across the entire CPF superfamily. The PHR domain is further divided into an N-terminal αβ subdomain and a C-terminal α-helical subdomain. These subdomains are connected by a long, flexible linker, and the C-terminal α-helical one hosts the binding pocket for the flavin adenine dinucleotide (FAD) cofactor, which acts as the light-absorbing chromophore common to all CPF members.^14^ Appended to the PHR is a C-terminal extension, or C-terminal tail (CTT), of variable length and sequence, which differs across species and is thought to confer functional specificity, for instance by mediating protein–protein interactions or modulating the lifetime of the photoactivated state.

**Figure 1:**
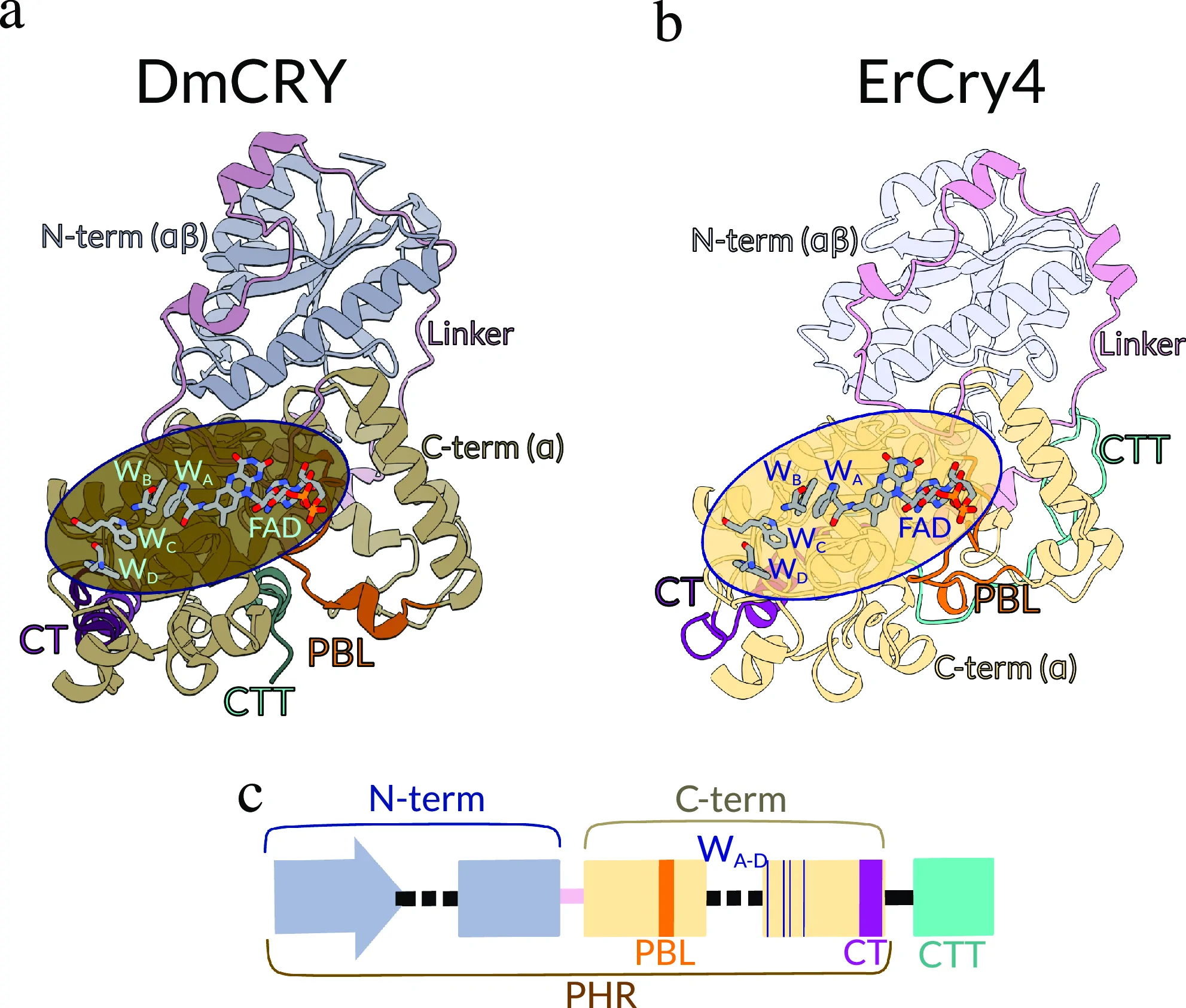
3D structure of putatively magnetosensitive animal cryptochromes with the photoactive core highlighted. (a) Crystal structure of DmCRY (PDB ID: 4GU5)^15^ (b) AlphaFold modeled and molecular dynamics-relaxed structure of full-length ErCry4 from ref 16. (c) Overall sequence of DmCRY and Cry4 highlighting the main domains, the phosphate-binding loop, and the C-terminal features. Notably, the C-terminal tail (CTT) of DmCRY’s crystal structure is more ordered – with a partially helical fold – than the modeled one of ErCry4.

The vast majority of cryptochromes are sensitive to blue light, thanks to the embedded FAD cofactor.^17–19^ FAD is a versatile redox-active chromophore that can exist in several oxidation and protonation states. Absorption of blue light promotes the oxidized flavin moiety to an electronically excited state, initiating the photochemical reactions that underlie cryptochrome function.^20^ The photochemistry of cryptochromes has been the subject of extensive investigation especially in plants,^12,21,22^ and has provided much of the understanding of flavin-based signaling. In plants, blue-light absorption by FAD triggers intraprotein electron transfer from a conserved tryptophan triad, generating a flavin semiquinone radical (FADH^•^) that drives conformational changes in the C-terminal extension and initiates downstream signaling cascades.

Among animal cryptochromes, avian Cryptochrome 4 (Cry4) has received particular attention as a potential magnetoreceptor. Its primary photochemical mechanism is now relatively well established and closely parallels that of plants. In Cry4, however, the tryptophan chain is extended by one additional residue to form a tetrad (W_A_-W_D_). Upon excitation, the flavin (FAD*) acts as an electron acceptor and abstracts an electron from the nearest tryptophan residue, W_A_, generating the first charge-separated radical pair, [FAD^•–^ W_A_^•+^]. The positive charge (hole) then sequentially migrates along the tryptophan tetrad, producing radical pairs with progressively larger charge separations. These radical pairs of the type [FAD^•–^ W^•+^] are known as RP1. RP1 then relaxes through protonation of FAD^•–^ and deprotonation of W^•+^ to generate the neutral radical pair RP2 (Figure 2).

**Figure 2:**
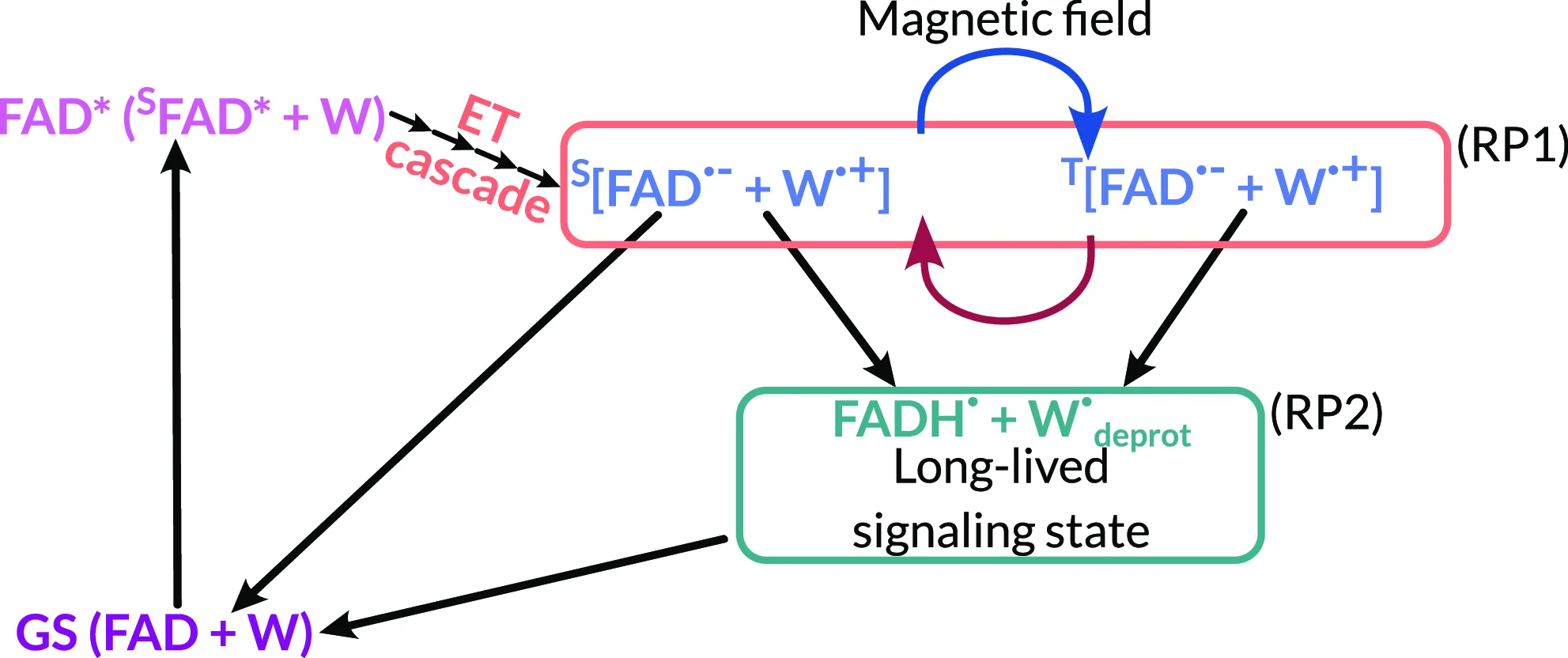
Scheme of the radical pair formation in cryptochromes. From the oxidized ground state (GS), FAD is excited by light absorption to a FAD* state. Subsequently, the ET cascade produces RP1 intermediates that interconvert between their singlet and triplet states at a rate depending on the magnetic field. These intermediates finally evolve to the RP2 state, with the singlet ones being moreover able to revert to the GS.

These photoinduced radical-pair states constitute the basis of the radical-pair mechanism (RPM) proposed for magnetic field sensing in cryptochromes.^23^ The RPM rests on the quantum mechanical principle that, following electron transfer, the radical pair undergoes interconversion between its singlet (S) and triplet (T) spin states (Figure 2). This interconversion is sensitive to the orientation and strength of external magnetic fields, including the geomagnetic field. Because singlet pairs can recombine to reform the ground state, while triplet pairs are longer-lived, the Earth’s magnetic field can shift the S/T population balance and thereby modulate the yield of the more long-lived radical pair state RP2 (Figure 2), and ultimately of downstream signaling products. This field-dependent yield, averaged over the orientations sampled during the birds’ head movements, could in principle provide directional information, effectively acting as an inclination compass.^23^

According to the RPM, the magnetosensitive capacity of Cry4 can thus be divided in three steps: (1) the photochemical step, in which FAD’s light absorption triggers a hole-hopping cascade along the tryptophan tetrad; (2) the spin-dependent step, in which the resulting radical pair interconverts between singlet and triplet states under the influence of the magnetic field; and (3) the structural step, in which the transient information carried by the radical pair is converted into a long-lived signaling state that activates downstream interactions. These steps depend on one another. The photochemistry determines which radical pair forms and how long it lives. The spin dynamics determine whether that pair can encode a magnetic signal. The conformational response determines whether the signal is transduced before it decays. The electron-transfer cascade of step (1) is now described with growing confidence, although the identity and stability of the final radical pair are still debated. Step (2) is, from a physical standpoint, the least established part of the mechanism: neither experiments nor calculations have yet shown conclusively that radical-pair spin dynamics are sensitive enough to explain magnetoreception under physiological conditions.^24^ Finally, the structural dynamics of step (3) after activation remain largely unresolved.

In this perspective, we examine what computational simulations have revealed about each of these steps. Our focus is on vertebrate Cry4, but we take the cryptochrome of *Drosophila melanogaster* (DmCRY) as a starting point, since it is by far the best characterized animal cryptochrome, both structurally and functionally.

## Lessons from *Drosophila* Cryptochrome

The ability to detect weak external magnetic fields is not restricted to migratory species. Magnetic-field-dependent behavioral responses have been reported in *Drosophila* and several other nonmigratory animals expressing cryptochromes,^25–27^ suggesting that magnetosensitivity may arise from fundamental physicochemical properties shared among these flavoproteins. In particular, the propensity of flavin cofactors such as FAD to generate photoinduced radical pairs provides a common molecular basis for magnetic-field effects. In migratory animals, including birds, this intrinsic sensitivity is thought to have been evolutionarily optimized to support directional navigation, whereas the underlying cryptochrome-dependent photochemistry appears to be broadly conserved across both navigating and non-navigating species.

Although *D. melanogaster*’s cryptochrome (DmCRY) structurally and functionally differs from avian Cry4, computational studies of this protein have provided important conceptual insights into the molecular mechanisms linking flavin photochemistry, conformational activation, and magnetosensitivity. In particular, a series of recent investigations has progressively clarified how photoinduced electron transfer generates long-range structural responses and, at the same time, has expanded the range of possible magnetoreception mechanisms beyond the canonical radical-pair paradigm.

A major advance in this direction was provided by Wang et al.,^28^ who investigated the molecular origin of DmCRY photoactivation through extensive molecular dynamics (MD) simulations, free-energy calculations, adaptive and enhanced sampling MD. Two competing mechanisms had been proposed for DmCRY activation. The first involved the canonical reduction of the oxidized flavin cofactor, FAD_ox_, through electron transfer along the conserved tryptophan triad/tetrad, leading to formation of the anion radical FAD^•–^. The second proposed alternative ET pathway involved other donors, including the adenine moiety of FAD itself. To discriminate between these possibilities, the authors investigated three different redox states of the cofactor: FAD_ox_, FAD^•–^, and an intramolecular charge-transfer state involving the adenine moiety. Their simulations demonstrated that only the formation of FAD^•–^ can trigger the large-scale conformational changes associated with activation. In particular, photoreduction of FAD destabilizes a conserved salt bridge (R381-D410) located near the flavin-binding pocket, lowering the free-energy barrier for its dissociation and identifying this interaction as a redox-sensitive allosteric switch. Disruption of the salt bridge reorganizes the hydrogen-bonding network connecting the N- and C-terminal regions, ultimately promoting release of the autoinhibitory C-terminal tail (CTT) and initiating its helix-to-coil transition, as previously observed with proteolysis, spectroscopic and time-resolved X-ray scattering measurements.^29–31^ This work therefore established a direct mechanistic connection between flavin photoreduction and long-range conformational activation, providing one of the first atomistic descriptions of signal transduction in cryptochromes. This proposal found an experimental confirmation in the structural resolution of the activated DmCRY interacting with its target protein Timeless (TIM),^32^ which, accordingly, no longer presents the R381-D410 salt bridge.

Building on the framework of Wang et al.,^28^ Chandler et al.^33^ combined MD simulations with hydrogen-deuterium exchange mass spectrometry (HDX-MS) and cavity ring-down spectroscopy to experimentally characterize the structural consequences of photoactivation. Their simulations identified early light-induced rearrangements of the phosphate-binding and protrusion loops surrounding the flavin cofactor, suggesting that these local motions represent the first step of the activation process. HDX-MS measurements subsequently revealed that these local perturbations propagate to distant regions of the protein, particularly C-terminal helices (α22 and α23), which become significantly more solvent-exposed upon blue-light illumination. The resulting conformational state persists much longer than the lifetime of the primary radical-pair intermediates, and therefore constitutes a plausible signaling state. Importantly, mutation of the terminal tryptophan of the ET chain abolished most of the large-scale structural response, demonstrating that conformational activation is directly coupled to tryptophan-mediated photoreduction of FAD. Together with the earlier work of Wang et al., these results support a unified picture in which the formation of FAD^•–^ triggers local structural rearrangements around the flavin that are subsequently amplified through long-range allosteric pathways, culminating in the release of the C-terminal signaling region.

These two studies reinforce the canonical view that activation originates from the FAD+4W ET chain. Other experiments, however, indicate that this chain is not the only route available. Mutation of the tryptophan chain does not abolish phototransduction in DmCRY or in other cryptochromes,^34–36^ and in the animal-like cryptochrome of *C. reinhardtii* a secondary ET pathway has been identified, running in the direction opposite to the primary one.^37^ Along the same line, recent studies by Bradlaugh et al.^27,38^ showed that the isolated C-terminal tail of DmCRY, which lacks both the photolyase homology region and the conserved tryptophan tetrad, can nevertheless support magnetic responses. Combining MD simulations with biochemical and electrophysiological measurements, they identified previously unrecognized FAD-binding sites within the intrinsically disordered C-terminal region. The simulations revealed two stable electrostatically-driven binding modes that stabilize FAD in an unusually extended conformation characterized by an increased separation between the isoalloxazine and adenine moieties. Such conformations have been proposed to favor long-lived intramolecular radical pairs and thereby enhance magnetic field sensitivity. Experimental mutagenesis confirmed the importance of these electrostatic interactions for magnetic responsiveness. These findings led the authors to propose a noncanonical mechanism in which the C-terminal tail itself acts as a flavin-binding magnetic sensor and signal transducer. Although still debated, this work significantly broadens the mechanistic landscape of cryptochrome-mediated magnetoreception by suggesting that magnetically sensitive flavin states may arise outside the canonical FAD-binding pocket and independently of the conserved tryptophan ET chain.

All these studies illustrate how computational approaches have progressively expanded our understanding of cryptochrome function, from the identification of allosteric pathways linking flavin photoreduction to conformational activation, to the characterization of long-lived signaling states, and finally to the exploration of alternative architectures for magnetic sensing. Although these studies were performed on DmCRY, they provide valuable conceptual frameworks for current investigations of avian Cry4, where the molecular connection between radical-pair formation, magnetic sensing, and signal transduction remains an unresolved challenge.

## Avian Cryptochrome 4

Birds possess three cryptochrome genes: Cry1, Cry2 and Cry4. Like their mammalian orthologs, Cry1 and Cry2 seem to have lost a stable FAD binding^39,40^ and to be involved in circadian timing,^39^ as their daily expression oscillations suggest.^41^ Cry4, by contrast, reaches peak expression during the migration season^41^ and noncovalently binds FAD in a pocket between the N-terminal and C-terminal domains. These features make Cry4 the most plausible magnetoreceptor candidate among avian cryptochromes.

Although Cry4 is also found in fish, amphibians and reptiles,^14,42^ it has been studied mainly in birds and, especially, in the night-migratory European robin (*E. rubecula*). Two Cry4 isoforms exist in this species, ErCry4a and ErCry4b, but *in vitro* measurements and interaction-energy calculations indicate that only ErCry4a binds FAD.^43^ We therefore use ErCry4 to mean ErCry4a throughout this text. Recent *in vitro* absorbance measurements of the photoinduced radicals have shown that ErCry4 is more sensitive to a 10 mT magnetic field than its orthologs from pigeon (ClCry4) and chicken (GgCry4).^13^

Although ErCry4 has been extensively studied by spectroscopy,^13,44^ no experimental structure of this protein is available. Computational studies of ErCry4 therefore start from the crystal structure of its closest homologue with a solved structure, ClCry4 (PDB ID: 6PU0),^45^ which shares 87.9% sequence identity with ErCry4.^16^ Even the ClCry4 structure is incomplete: it lacks two disordered, flexible segments, the phosphate-binding loop (PBL, residues 232–249) and the ∼30-residue C-terminal tail (CTT, residues 498–526).^45^ As in DmCRY, both regions have been linked to the structural transduction of the magnetic signal in Cry4, experimentally^46^ and computationally.^45,47,48^ Figure 1 highlights them in the crystal structure of DmCRY^15,49^ and in an AlphaFold model of ErCry4 relaxed by molecular dynamics (MD).^16^

In the next sections, we are going to follow (1) the photoactivation with the ET cascade; (2) the radical pair mechanism; and (3) the structural transduction. For each step, we highlight where computational and experimental results agree and identify the gaps that remain to be closed.

### The ET Cascade

In ErCry4, the ET cascade involves four tryptophan residues, W395 (W_A_), W372 (W_B_), W318 (W_C_) and W369 (W_D_), whose roles have been confirmed by single-point mutations.^13^ In the crystal structure of ClCry4,^45^ as in the modeled structures of ErCry4 and other orthologs,^13,16,50,51^ this chain extends from the FAD binding pocket to the protein surface, where the W_D_ radical can be stabilized by the solvent.^51^ Upon photoexcitation, FAD is reduced by W_A_, forming the first radical pair, RP_A_ = [FAD^•–^W_A_^•+^]. The hole then hops along the chain, giving rise to the successive radical pairs RP_B_, RP_C_ and RP_D_. In each of these, FAD is a radical anion (FAD^•–^) and one tryptophan is a radical cation.

Biological electron transfer is commonly described with Marcus theory, which treats charge transfer as a transition between charge-localized initial (*i*) and final (*f*) states. For each hole-hopping step in Cry4, the initial state is [FAD^•–^ W_*n*_^•+^] and the final state is [FAD^•–^ W_*n*+1_^•+^]. In its original formulation, Marcus theory gives the transfer rate as

1 $$k_{i\to f}=\frac{|V_{\mathrm{if}}{|}^{2}}{\hbar }\sqrt{\frac{\pi }{\lambda_{\mathrm{if}}k_{B}T}}\exp\left(-\frac{{\left(\Delta G_{\mathrm{if}}^{{}^{\circ}}+\lambda_{\mathrm{if}}\right)}^{2}}{4\lambda_{\mathrm{if}}k_{B}T}\right)$$

which depends on three quantities: the electronic coupling *V*_*if*_, the reorganization energy λ_*if*_, and the driving force Δ*G*_*if*_^°^. The driving force is the free-energy difference between the final and initial states, which determines the ratio of forward and backward transfers according to the detailed balance relationship:

2 $$\frac{k_{i\to f}}{k_{f\to i}}=\exp\left(-\frac{\Delta G_{\mathrm{if}}^{{}^{\circ}}}{k_{B}T}\right)$$

The reorganization energy λ_*if*_ is the free-energy cost of displacing the nuclei from the equilibrium configuration of state *i* to that of state *f* while the system remains in state *i*. Within linear response, both quantities follow from the statistics of the vertical energy gap Δ*E*_*if*_ = *E*_*f*_ – *E*_*i*_, which depends on the nuclear configuration:

3 $$\Delta G_{\mathrm{if}}^{{}^{\circ}}=\frac{1}{2}\left({\left\langle \Delta E_{\mathrm{if}}\right\rangle }_{i}+{\left\langle \Delta E_{\mathrm{if}}\right\rangle }_{f}\right)$$

4 $$\lambda_{\mathrm{if}}=\frac{1}{2}\left({\left\langle \Delta E_{\mathrm{if}}\right\rangle }_{i}-{\left\langle \Delta E_{\mathrm{if}}\right\rangle }_{f}\right)$$

where ⟨Δ*E*_*if*_ ⟩_*x*_ is the average gap over the nuclear ensemble equilibrated with state *x* = *i*, *f*. Physically, the electron is transferred at a fixed nuclear configuration. The nuclei then relax on the free-energy surface of the final state, which lowers the free energy by λ_*if*_, so that Δ*G*_*if*_^°^ = ⟨Δ*E*_*if*_ ⟩_*i*_ – λ_*if*_. Further details can be found in ref 52.

Computing Δ*G*_*if*_^°^ and λ_*if*_ therefore requires sampling of both the initial and the final state. This sampling is usually performed with classical MD simulations. With a molecular mechanics (MM) force field, the two states differ only in the partial charges assigned to the donor and acceptor moieties. Electronic couplings, by contrast, generally require quantum mechanical (QM) calculations.^52,53^ One of the most cost-effective schemes is the frontier-orbital approximation of Troisi and Orlandi.^54^

Standard Marcus theory rests on several assumptions. First, the electronic coupling *V*_*if*_ is taken to be weak, so that the charge stays localized and hops nonadiabatically from one site to the next. Second, the energy-gap distribution is taken to be Gaussian. This is equivalent to linear response, and it yields eqs 3 and 4 as well as parabolic free-energy surfaces of equal curvature.^52^ Third, and more subtly, nuclear relaxation is taken to be much faster than electron transfer. In other words, the chromophore, the protein and the surrounding water are assumed to re-equilibrate faster than the hole hops. Finally, the nuclei are treated classically.

Having outlined the Marcus framework and its limits, we now turn to how it has been applied to the ET cascade of Cry4. *In vitro* measurements on avian cryptochromes have shown that ErCry4 responds more strongly to a magnetic field than ClCry4.^13^ Because the magnetic response depends on the radical pair produced at the end of the ET cascade, a natural question is whether this difference can be traced back to the cascade itself. Simulations have tackled this question in two ways. Most studies estimate the parameters of eq 1 for each hop and compute rates and equilibria from them. Others simulate the hole transfer in real time, which removes some of the assumptions of Marcus theory discussed above.

Following the first strategy, Xu et al.^13^ used MD simulations of a homology model of ErCry4 to estimate the Marcus parameters for each hole-transfer step. Their results suggested that RP_C_ and RP_D_ are in equilibrium, with RP_C_ more populated, so that the hole would reside mainly on W_C_. Hanic et al.^50^ then compared ErCry4 with other avian Cry4 proteins by MD and found very similar structural dynamics across species. They also noted, however, that homology-modeled structures showed somewhat larger fluctuations in the C-terminal region, even though the C-terminal tail had been removed. As discussed below, this sensitivity to the starting model turns out to be central.

Cascone et al.^16^ addressed the ErCry4/ClCry4 difference directly. They started from AlphaFold3 ^55^ models of both proteins and combined MD sampling with QM/MM calculations of the energy gaps and couplings. Two results stand out. First, the electronic coupling of each hop depends strongly on the relative orientation of the two residues, not just on their distance. Because the rate scales with |*V*_*if*_|^2^, small geometric changes can therefore have a large effect on the kinetics. Second, the free-energy ladder of the RP states differs only slightly between ErCry4 and ClCry4, which suggests that the ET cascade is highly conserved among avian Cry4 proteins. Unlike Xu et al., however, these calculations favored RP_D_ over RP_C_ as the final state in both species.

Experiment cannot yet settle this disagreement. The simulated kinetics of ref 16 agree well with transient absorption spectroscopy,^44^ but transient absorption cannot distinguish RP_C_ from RP_D_ and therefore says nothing about which is more stable. The EPR measurements of Xu et al.^13^ give a radical-pair distance consistent with a predominant RP_D_ population. These measurements were, however, performed at cryogenic temperatures (80 K), where the equilibrium may differ from that at physiological temperature. For now, the position of this equilibrium can only be inferred from calculations.

Within Marcus theory, the equilibrium depends only on Δ*G*_CD_^°^ through detailed balance (eq 2). Because *k*_B_ *T* ≈ 0.6 kcal/mol at room temperature, a free-energy difference below 1 kcal/mol is enough to shift the population from one state to the other. The discrepancy between refs 13,16 lies precisely in this quantity. Both studies obtained it from MD sampling, but only ref 16 computed the energy gaps with QM/MM. Nevertheless, the key difference turned out to be the starting structure rather than the level of theory: in fact, QM/MM calculations on the MD trajectories of ref 13 also gave a positive Δ*G*° for the RP_C_ → RP_D_ hop (0.4 kcal/mol).^16^

Luo et al.^51^ later reached the same conclusion from a different angle. Starting again from a homology model of ErCry4 and combining MD with QM/MM, they found RP_C_ and RP_D_ to be almost isoenergetic, with RP_C_ favored by only 0.2 kcal/mol. This value is close to the 0.4 kcal/mol that Cascone et al. obtained from the trajectories of ref 13. Taken together, these studies show that the predicted free-energy differences depend strongly on the initial structural model, but only weakly on the QM method or the QM/MM scheme. Homology models of cryptochromes should therefore be built and validated with particular care.

Luo et al. also examined what controls these free-energy differences. They decomposed the electrostatic potential at the donor and acceptor sites into contributions from individual residues and from the solvent. Since this potential is what shifts the energy gap Δ*E*_*if*_, it directly determines both Δ*G*_*if*_^°^ and λ_*if*_ (eqs 3–4). Surrounding water emerged as the dominant factor stabilizing both RP_C_ and RP_D_ relative to the neutral state, with a far larger effect than the protein. The protein instead fine-tunes the RP_C_/RP_D_ balance through a few residues. For example, replacing the positively charged K320 with a neutral residue would change the electrostatic potential between W_C_ and W_D_ and thus shift the equilibrium. Notably, K320 is one of the few positions where ErCry4 and ClCry4 differ: ClCry4 carries a glutamate there, which reverses the charge (see the Supporting Information of ref 16 for the full sequence alignment). Whether this substitution contributes to the different magnetic responses of the two proteins remains to be tested.

All of these studies, however, rely on the assumptions of Marcus theory. Timmer et al.^44^ instead followed the second strategy and simulated the hole-hopping cascade of ErCry4 in real time with many independent nonadiabatic QM/MM MD trajectories. To reach nanosecond timescales at an affordable cost, they used the semiempirical DFTB method together with the fragment molecular orbital approximation.^56^ In principle, this approach removes two of the Marcus assumptions discussed above: that the charge stays strictly localized, and that nuclear relaxation is much faster than charge transfer. The nuclei, however, are still treated classically.

These simulations predicted only a small hole population on W_C_ and W_D_, which the authors attributed to incomplete statistical convergence. When they restricted the analysis to trajectories that did reach RP_C_ or RP_D_, the results agreed qualitatively with the transient absorption kinetics. In this subset, RP_C_ remained only slightly populated, whereas RP_D_ became the main state after about 500 ps. This picture is closer to the RP_D_-favored equilibrium of ref 16.

Comparing the two strategies with experiment for each step also reveals the timescales of the cascade. Overall, the forward hops become progressively slower along the tetrad (Figure 3). Hole transfer from the locally excited FAD to form RP_A_ takes ≤ 1 ps.^16,44^ The subsequent steps occur on the picosecond-to-nanosecond timescale.

**Figure 3:**
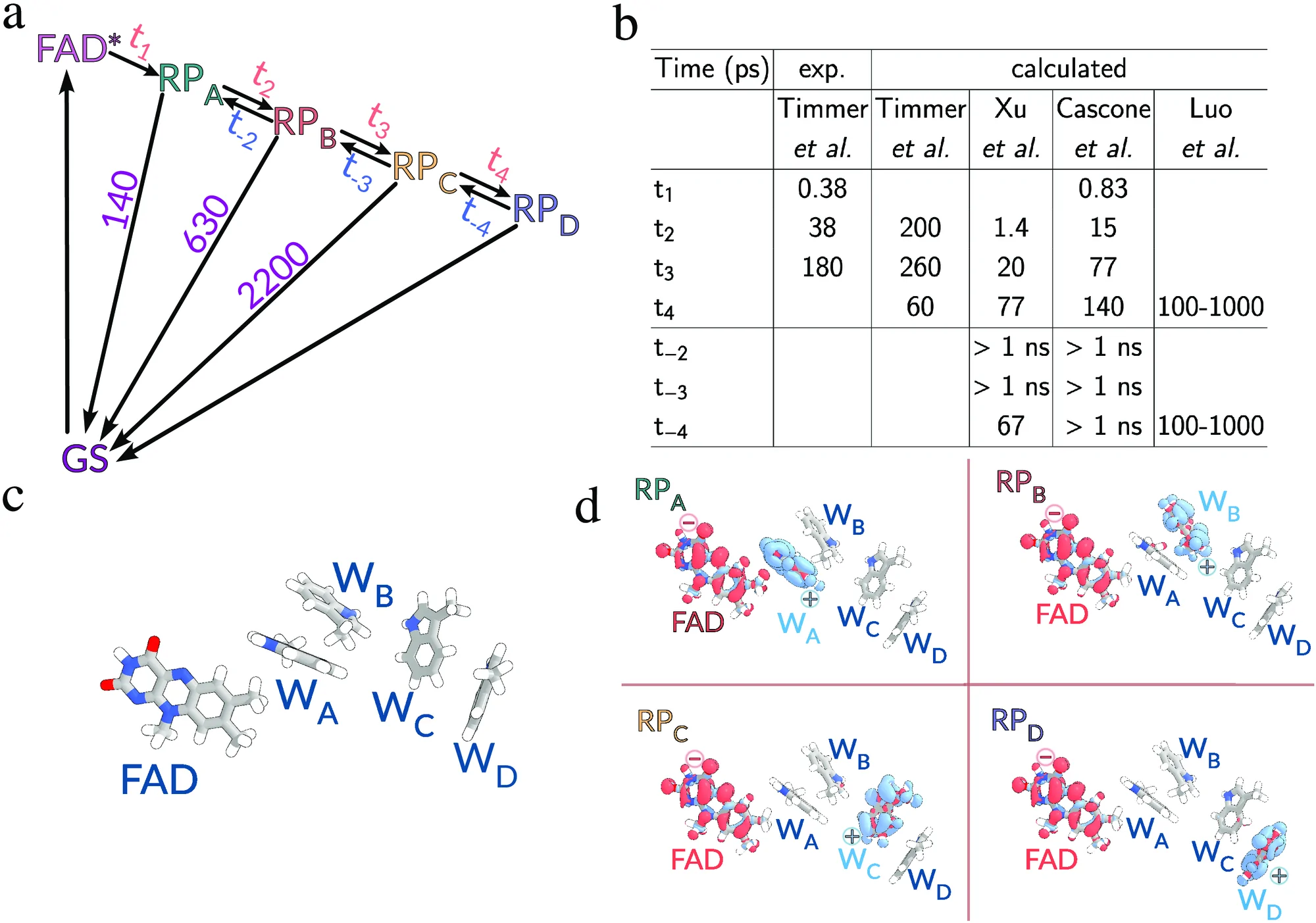
Summary of the ET cascade in ErCry4. (a) Scheme of the ET steps. The times *t*_1_..._4_ refer to the inverse rates of forward hole transfer, whereas *t*_–1_..._–4_ are the inverse rates of back-transfer. The recombination rates (RP_X_ → GS) are those extracted from the experiments.^44^ (b) Summary of experimental and calculated^13,16,44,51^ ET rates. (c) Arrangement of FAD with the tryptophan tetrad. (d) Depiction of the radical pair states.

For the RP_A_ → RP_B_ step, transient absorption gives an observed time constant of 30 ps in wild-type ErCry4, which reflects competition with charge recombination to the ground state.^44^ From the ∼78% yield of this step, the forward rate is (38 ps)^−1^ and the recombination rate is (140 ps)^−1^. Marcus-based calculations predict 1.4–15 ps for this step,^13,16^ whereas the nonadiabatic simulations give ∼200 ps.^44^

The RP_B_ → RP_C_ step is slower in both experiments and calculations. The experimental data imply a rate of (180 ps)^−1^.^44^ Marcus-based estimates range from 20 ps^13^ to 77 ps,^16^ while the nonadiabatic simulations predict 260 ps.^44^ For both of these steps, the Marcus estimates are faster than experiment and the nonadiabatic estimates are slower. Plausible explanations are, respectively, the strong dependence of |*V*_*if*_|^2^ on the sampled geometries and the limited statistics of the real-time trajectories.

The RP_C_ ⇄ RP_D_ transfer has not been resolved experimentally. Rate calculations place it between 100 ps and 1 ns, slightly slower than the preceding steps.^16,51^ Depending on the sign and size of Δ*G*_CD_^°^, the back-transfer is either comparable to the forward rate^51^ or negligible.^16^ The nonadiabatic simulations instead predict a much faster transfer, of about 60 ps.^44^

Finally, a very recent preprint by Hungerland et al.^57^ extended the nonadiabatic approach of Timmer et al. by explicitly including the vibrational degrees of freedom of the tryptophans. This extension improved the agreement with spectroscopic observations, but it led to a markedly different conclusion on the relative populations of RP_C_ and RP_D_. These results suggest that one of the central assumptions of Marcus theory, namely that the nuclei fully re-equilibrate before each hop, should be applied with caution to the Cry4 cascade. They also show that the RP_C_/RP_D_ equilibrium, and hence the identity of the magnetosensitive radical pair, remains an open question.

### The Radical Pair Mechanism

The magnetosensitivity of cryptochromes depends on the fact that the product of the ET cascade is a spin-correlated radical pair, which can interconvert between singlet and triplet states (see Figure 2). Only the singlet state can revert to the GS, while both states can evolve to form the RP2 intermediate. The ratio between these two channels, and thus the yield of their products – the recombined GS and RP2 – depends on the singlet-triplet interconversion that occurs while the electron spins in the radical pair are correlated.^23^

The radical pair mechanism can be described within a model that considers few key interactions involving the unpaired electrons. The radical pair RP1 (see Figure 2) is modeled by the Hamiltonian^58,59^

5 $${Ĥ}_{\mathrm{RP}}={Ĥ}_{\mathrm{ex}}+{Ĥ}_{\mathrm{dip}}+{Ĥ}_{Z}+{Ĥ}_{\mathrm{hf}}$$

where *Ĥ*_ex_ and *Ĥ*_dip_ respectively contain the exchange and dipolar interactions between the two spins, *Ĥ*_*Z*_ is the Zeeman Hamiltonian for the interaction between each spin and the external magnetic field, and *Ĥ*_hf_ is the hyperfine coupling Hamiltonian. The spin state of RP1, initially a singlet, evolves in time according to *Ĥ*_RP_.

In the minimal radical-pair model considered here, the exchange and dipolar interactions only modify the relative energies of singlet and triplet states. Instead, the term that can mix singlet and triplet states is the hyperfine interaction *Ĥ*_hf_, which acts as a driver of *S* ↔ *T* interconversion.^23^ With these interactions, an initially populated singlet state evolves toward a triplet state, and vice versa. The resulting oscillations (represented schematically by the curved arrows in Figure 2) have an oscillation period that depends on the energy differences between eigenstates. The Zeeman Hamiltonian *Ĥ*_*Z*_ is the only term that depends on the external magnetic field, and splits the triplet populations according to their magnetic quantum number. This alters the energy differences among all states, and thus modulates the *S* ↔ *T* oscillations.

Notably, the hyperfine interactions couple electronic spins to the nuclear spins of magnetic isotopes (e.g., ^1^H and ^14^N). In a minimal model, only one or few nuclear spins are considered.^58^ Importantly, this interaction is anisotropic and has a preferential direction that depends on the molecular orientation. On the other hand, the Zeeman interaction depends on the magnetic field orientation. It is then clear that the relative orientation between molecules and magnetic field can alter the oscillation of the singlet/triplet population and ultimately the yield of the spin-dependent reactions.^23^

Calculations with this model have shown that the yields of the spin-dependent reactions (recombination and evolution toward RP2) can be modulated by the magnetic field, under some assumptions.^23,59,60^ First, the two reactions must have similar rates. Second, the combined effect of exchange and dipolar coupling should be small enough to keep singlet and triplet states close in energy.^58^ This is possible considering that the two spins are spatially well separated.^59^ Third, the radical-pair lifetime must be at least equal to the Larmor period of ∼700 ns corresponding to the Earth’s magnetic field.

Another limit comes from the spin relaxation timescale, that is the time it takes for the spin state populations to approach thermal equilibrium, where the two spins are essentially uncorrelated. Population oscillations are damped on this timescale even if the radical pair itself is long-lived, and cannot be modulated by the magnetic field. Therefore, spin relaxation should also be slower than ∼700 ns.

It is important to note that the model outlined above assumes an overall unitary spin dynamics governed by the Hamiltonian *H*_RP_. In a realistic scenario, this Hamiltonian fluctuates in time due to vibrational and conformational motion in the protein.^61^ As a result, the spin system made by the radical pair and coupled nuclei becomes coupled to the protein motion as well. This coupling induces both spin relaxation and decoherence.^23,62^ The latter effect reduces the correlation between the two spins, even without relaxation, and effectively dampens the *S* ↔ *T* oscillations. Adams et al. introduced an open quantum systems perspective to this problem,^62^ considering what would be the effect of spin-protein couplings. They suggested that in realistic conditions decoherence effects would not significantly alter the spin dynamics of the radical pair.

Given their primary importance in the radical pair mechanisms, accurate estimates are needed for hyperfine couplings. These can be obtained from quantum chemical methods applied to FADH and tryptophan radicals. According to DFT calculations, for FADH, the nitrogens in the central ring, N5 and N10, show the largest hyperfine couplings with the radical.^63^ More recently, Deviers et al. calculated hyperfine couplings of FAD^–^ and FADH^•^ along a MD simulation of ClCry4 using hybrid QM/MM methods.^64^ Their results suggested that the average couplings do not significantly deviate from those derived from a static structure. However, they also revealed significant coupling fluctuations in the picosecond–nanosecond time scale due to protein motion, which could influence spin decoherence. Fluctuations of hyperfine couplings are in the order of 5–10 MHz, not negligible compared to the magnitude of hyperfine couplings (about 10–30 MHz for isotropic couplings).

To study the impact of coupling fluctuations on the spin dynamics, MD-based fluctuations have been included in open quantum systems treatments.^65,66^ These works showed that coupling fluctuations significantly suppress the quantum yield of the singlet reactions, as well as the sensitivity to the magnetic field orientation. These results highlight the importance of considering spin relaxation and decoherence mechanisms for a realistic modeling of the radical pair mechanism.

It is important to note that fluctuations occurring on timescales comparable to the radical-pair lifetime, i.e., approximately 0.1–1 μs, are expected to have a direct impact on the spin dynamics. MD simulations currently employed to parametrize the spin Hamiltonian typically span sub-μs time scales.^64,65^ It is therefore still uncertain whether these simulations fully capture the low-frequency motions that may dominate spin relaxation and decoherence in cryptochrome.

Including more realistic interactions, as done in more recent work, appears to reduce the orientational sensitivity of the radical pair mechanism.^67^ The results of ref 67 show that coupling fluctuations may enhance the orientational dependence of the singlet yield. However, the inclusion of hyperfine couplings with more nuclear spins dramatically decreases the effect. As a consequence, it appears that more realistic models result in very small orientational dependence at geomagnetic field intensities.^67^

The latter results are in agreement with the magnetic field strengths of 10 mT necessary to observe an effect in ErCry4,^13^ orders of magnitude larger than the geomagnetic field (∼50 μT). This remains the main limitation of the radical pair mechanism, and highlights the need of realistic modeling for the magnetic sensitivity of bird Cry4. Very recently, a revised model was proposed, which combines the radical pair mechanism with magnetic field enhancement arising from magnetite crystals.^68^

### Structural Transduction

The structural characterization of photoactivated Cry4 has been so far complicated by the lack of experimental information. Indeed, no crystal structure exists for avian Cry4s in their activated form, neither in isolation nor in complex with potential signaling partners. The only experimental reference is the crystal structure of ClCry4,^45^ in its dark-adapted state with oxidized FAD. Proteolysis assays coupled to mass spectrometry on the same protein nonetheless indicated that blue-light illumination alters the accessibility of the PBL and the CTT (Figure 1),^45^ which suggests that both regions change conformation upon photoactivation.

In the absence of a structure of the signaling state, simulations have been the main tool for following these changes, mostly in ErCry4.

From computational works, the PBL emerges as a gating element that senses the redox state of the flavin. All-atom MD simulations of ClCry4 on the microsecond timescale, run for both GS and RP_D_, indicate that the loop can open in RP_D_, exposing FAD to the solvent.^47^ The same behavior appears when an entire redox cycle is simulated with a coarse-grained (CG) model.^69^ There, the PBL opens in RP_D_ and closes again in the subsequent RP2_D_ state, [FADH^•^ W_D,deprot_^•^], in which the flavin has been protonated and W_D_ deprotonated. This succession can be interpreted as functional to the access of water to FAD’s anionic radical and to its subsequent protonation.

Two aspects limit the reach of this picture: the timescales involved and the starting model. As explained in the previous section, the radical pair lifetime should be at least 700 ns.^23,70^ Conventional all-atom simulations reach comparable or shorter times, so only the longer trajectories accessible to coarse-grained models can follow the signaling response as it unfolds. The PBL, moreover, is unresolved in the crystal structure and, in the above-mentioned works, was rebuilt using homology modeling. Within the ground state, simulations can lead to an alternative conformation of the loop that is more stable than the initially modeled one and from which no opening occurs.^47^

The PBL is not the only region that responds to photoactivation. When an equilibrated ground-state configuration is switched to RP_C_ by an instantaneous charge redistribution, CG-MD replicas show a displacement of the C-terminal region (CT, residues 470–497, purple in Figure 1) and of the EEE region that precedes it.^48^ The latter contains a conserved motif of three glutamates which, in DmCRY, has been proposed to act as a recognition site for partners carrying PDZ domains.^27,48^ Because these displacements are small and involve disordered regions, they are detected by comparing the conformational distributions sampled in the different states rather than by inspecting individual structures.^48^ The same analysis links the CT region to the tryptophan chain: changes in its conformation correlate with changes in the positions of W_C_ and W_D_ relative to FAD.

The PBL, CT and EEE regions respond to the GS → RP_C_ switch with different time constants, and the PBL is the slowest. At first sight this conflicts with the PBL gating reported above, which was seen in simulations of RP_D_ that were in part shorter. A more likely explanation is that the loop motion of ref 47. accompanies the evolution of the system into RP_D_ rather than the formation of RP_C_.

All of these results share one limitation: they were obtained on a truncated ClCry4 structure that lacks the CTT. Although this simplification does not invalidate the PBL gating mechanism, it leaves out a region that is disordered, hard to model, and widely held to be central to photoactivation and signal transduction in DmCRY.^28,38^ In avian Cry4, the CTT has long been regarded as the likely site of interaction with signaling partners,^71^ and a recent study points to it as a possible site of interaction with the lipid bilayer of the cell membrane.^72^ Such an interaction, whether direct or mediated by partners, matters functionally: anchoring Cry4s in an ordered fashion within the cell is a prerequisite for sensing the direction of the field.^23,73^

Because the CTT is intrinsically disordered, there is no single obvious way to model it and different approaches have been used in the literature.^16,72,74^ Including a modeled CTT has already changed the picture. Simulations of the full-length protein reveal a structural correlation between the CTT and the PBL that appears stronger in ClCry4 than in DmCRY, and enhanced sampling MD simulations of the CTT conformational space point to several distinct conformational states of the protein.^75^

These predictions can be compared with two sets of experiments: the proteolysis assays on ClCry4 mentioned above^45^ and recent spectroscopic work on ErCry4.^46^ The latter used HDX-MS, the same approach applied earlier to the postactivation dynamics of DmCRY.^33^ Under continuous irradiation, three states of ErCry4 could be resolved: the GS, RP2 and the fully reduced state, in which FAD is present as FADH^–^. Each state showed a distinct structural signature, and the motions of the PBL and the CTT differed between them. These data provide independent evidence for the coupling between the two regions predicted by simulations,^75^ and indicate that in RP2 the PBL acts together with the protrusion loop. They also question the reclosing of the PBL in RP2, which had been obtained from simulations of the truncated protein.^69^

Together, these results indicate that the CTT must be modeled explicitly if the photoactivation of avian Cry4 is to be simulated correctly. Characterizing the postactivation structural behavior of the protein remains difficult, but simulations and experiments are converging on a consistent description. This description is also relevant to the downstream steps, since, as in DmCRY, the interaction of Cry4 with its cellular partners is generally assumed to be triggered by a conformational change.^14^ Six candidate partners have been identified experimentally:^71^ the long-wavelength-sensitive opsin (LWS), a voltage-gated potassium channel, a retinol-binding protein, a retinal G-protein coupled receptor, and the γ-10 and α-2 subunits of the cone-specific G­(t) protein. The last of these transduces signals in the visual pathway, and its interaction with ErCry4 has been confirmed by pulldown assays and FRET experiments in retinal cells.^76^ These results support the hypothesis that Cry4 shares part of the signaling machinery of vision at night. In the same experiments, the subunit was also found to bind a CTT-truncated form of ErCry4, which indicates an interaction mode that does not require the tail.

## Conclusions

The picture emerging from a decade of computational work is that of a field advancing unevenly along the three steps that link photon absorption to a cellular signal.

The early photochemical events are now understood with growing confidence. The hole-hopping cascade along the W_A_–W_D_ tryptophan tetrad has been characterized both through Marcus-theory rate calculations and through real-time nonadiabatic QM/MM dynamics, and the resulting radical-pair energy ladder is largely consistent across orthologs, supporting the view that the ET cascade is strongly conserved among avian Cry4. Even here, however, important questions remain open. The relative stability of RP_C_ and RP_D_, and thus the identity of the radical pair that ultimately leads to the magnetically sensitive signaling state, still depends sensitively on the starting structure and on the assumptions underlying the kinetic treatment.

The spin-dependent step instead still presents some critical aspects. Hyperfine couplings computed by QM/MM along MD trajectories agree on average with those from static structures, but they fluctuate on the picosecond–nanosecond time scale by a substantial fraction of their magnitude. When these fluctuations, and a realistic number of coupled nuclear spins, are introduced in the model, the predicted singlet yield and its orientational dependence are strongly suppressed, leaving the sensitivity at geomagnetic intensities very small. Supporting the magnitude of the effect at geomagnetic fields, whether through mechanisms internal to the protein or through hybrid schemes invoking magnetite-based amplification, remains a central open problem.

Finally, the structural transduction of the photoactivation signal remains the principal unresolved challenge. The conformational rearrangements that convert a transient radical pair into a long-lived signaling state involve intrinsically disordered regions that are difficult to resolve experimentally and computationally expensive to sample. The partial disagreements found in the literature indicate both the immaturity of the structural description and the importance of explicitly modeling the disordered C-terminal regions that earlier studies often truncated.

These three steps are not independent, and the most informative open questions lie at their interfaces: the protonation that converts RP1 into the long-lived RP2 state links the photochemistry to the structural response, and the identity of the assumed “final” radical pair (RP_C_ vs RP_D_) has an influence on the spin dynamics simulations. Likewise, the conformational dynamics of the protein controls the very fluctuations that govern spin relaxation. In a moment of unprecedented advances in protein structure determination, as well as in the prediction of protein complexes using AI-based approaches, a natural next step is to apply such methods to characterize the interaction interfaces of Cry4 and the conformations it adopts in association with its known partners. Combining these structural predictions with multiscale simulations of the photochemistry and of the spin dynamics offers a realistic route to connecting the building blocks of the signaling cascade to downstream cellular events.
